# The discrepancy of antemortem clinical diagnosis and postmortem autopsy diagnosis of lung pathologies in under-five deaths and the reasons for discrepancies: a case series analysis

**DOI:** 10.1186/s12887-024-04854-4

**Published:** 2024-05-28

**Authors:** Haleluya Leulseged, Chris A. Rees, Addisu Alemu, Beth A. Tippett Barr, Merga Dheresa, Lola Madrid, Anthony Scott, Nega Assefa

**Affiliations:** 1 Hararghe Health Research Partnership, Haramaya University, Harar Town, Jineala District, PO. BOX: 235, Diredawa, Ethiopia; 2 College of Health and Medical Science, Haramaya University, Diredawa, Ethiopia; 3 Division of Pediatric Emergency Medicine, Emory University School of Medicine, Atlanta, GA USA; 4 Nyanja Health Research Institute, Salima, Malawi; 5 London School of Hygiene and Tropical Medicine, London, UK

**Keywords:** Minimally invasive tissue sampling, Clinicopathologic discrepancy, Pathologic lung findings, Pathologic lung diagnosis, Child health, Diagnostic discordance

## Abstract

**Background:**

Diagnostic autopsy is the most reliable approach to definitively ascertain the cause of death and evaluate the accuracy of antemortem clinical diagnoses. Identifying diagnostic discrepancies is vital to understanding common gaps in antemortem clinical diagnoses and modifying antemortem diagnostic approaches to increase the accuracy of clinical diagnosis. The objective of this study was to determine the frequency of diagnostic discrepancies between antemortem clinical diagnoses and postmortem autopsies on lung pathologies and to understand the reasons for diagnostic discrepancies among cases included in Child Health and Mortality Prevention Surveillance (CHAMPS) in Ethiopia.

**Methods:**

A clinical case series study of deaths among children under-five in the CHAMPS study at three sites in Ethiopia between October 2019 and April 2022 was conducted. The antemortem clinical diagnoses and postmortem pathological diagnoses of the lung were compared for each case. Two senior physicians assessed the findings for both agreement and disagreement. McNemar’s test was used to assess for statistically significant differences between antemortem and postmortem diagnoses.

**Results:**

Seventy-five cases were included (73.3% male). Over half (54.7%) died between the 1st and 7th day of life. Sepsis (66.7%), pneumonia (6.7%), and meconium aspiration syndrome (5.0%) were the most common immediate causes of death. Half (52%) of cases were correctly diagnosed antemortem. The magnitude of diagnostic discrepancy was 35% (95% CI: 20-47%). The most common contributing factors to diagnostic discrepancy were gaps in knowledge (22/75, 35.5%) and problems in consultation and teamwork (22/75, 35.5%).

**Conclusions:**

Misdiagnoses were common among young children who died with positive lung pathology findings. In-service education initiatives and multidisciplinary collaboration are needed to mitigate high rates of diagnostic discrepancies among young children to potentially prevent future deaths.

## Background

Diagnostic errors contribute to as much as 10% of adverse events occurring in healthcare practice globally [1, 2]. This rate is higher in countries like Ethiopia, where there is limited diagnostic capacity and shortages of trained health professionals [3]. Diagnostic errors often result from diagnostic uncertainty, the complex nature of making accurate diagnoses in clinical practice, limitations in the availability and accuracy of some diagnostic tests, and time constraints encountered by clinicians [4]. Moreover, greater workload burdens, rising quality expectations, cognitive biases, and insufficient health system infrastructure may also contribute to diagnostic errors [5]. The increasing complexity in health care can be attributed to multiple factors such as a wide range of options for diagnostic testing and treatment, extensive and continually growing biomedical and clinical evidence influencing clinical practice, along with a higher occurrence of comorbidities among patients [6]. Diagnostic errors can lead to improper treatment and deaths that could potentially be averted if the correct diagnosis had been made [3].

Lung diseases, with their broad clinical and histological spectrum, present unique challenges as radiological and clinical signs are often non-specific [3]. Perinatal and under-five deaths due to lung diseases are a significant global burden leading to over 800,000 deaths each year among children aged < 5 years [7]. Detailed postmortem examinations of lung tissue yield the most accurate results when ascertaining the cause of death [8]. However, postmortem examinations are infrequently conducted in low- and middle-income countries, where the disease burden of lung pathology is highest among young children [9], leaving a gap in true understanding of causes of death and accuracy of antemortem clinical diagnoses for lung diseases.

Clinicopathologic discrepancy studies aim to evaluate the accuracy of clinical diagnoses by comparing them with the reference standard of postmortem autopsy diagnoses [10]. Despite advances in medical technology, significant discrepancies between antemortem clinical diagnoses and complete diagnostic autopsies (CDA) have been observed in as much as 45% of childhood deaths [10]. CDAs have long been considered the benchmark in establishing the cause of death (CoD). By conducting a comprehensive postmortem analysis, which involves examining internal organs and tissues, pathologists can gather insightful information regarding diverse diseases and their manifestations in the body, which can be applied to enhance the diagnosis, treatment, and prevention of these ailments for future patients.

CDA improves the identification of underlying causes and mechanisms of disease leading to death [8, 10]. However, the resources and expertise required for CDAs are often lacking in developing countries like Ethiopia [11–13]. Understanding which diagnoses are missed most often prior to a child’s death and the reasons for these diagnostic errors is crucial to identifying gaps in clinical diagnosis to refine diagnostic algorithms for improved treatment outcomes [3]. However, studies evaluating the accuracy of antemortem clinical diagnoses in Ethiopia are lacking but are essential to further guide policy and programs to reduce antemortem diagnostic errors, which, in turn, may reduce childhood mortality in regions with high rates of childhood mortality.

As an alternative to CDA, minimally invasive tissue sampling (MITS) has emerged as a viable method for ascertaining causes of death [11, 13], and has demonstrated high concordance with CDA [14]. Due to its acceptability and feasibility in resource-limited settings, MITS has been implemented by the Child Health and Mortality Prevention Surveillance (CHAMPS) network in nine countries across sub-Saharan Africa and South Asia.

Given the high burden of disease of lung diseases among young children and prior studies suggesting high rates of diagnostic errors in similar settings, our objective was to determine the frequency of, and reasons for, diagnostic discrepancies between antemortem clinical diagnoses and postmortem causes of death determined by lung pathology, in order to provide insight for health professionals and policymakers in the goal of reducing childhood mortality.

## Methods

### Study area and design

The study was conducted within the Health and Demographic Surveillance Sites (HDSS) areas of Kersa, Harar, and Haramaya, under Haramaya University in Ethiopia, the largest HDSS site in Africa [15, 16]. A clinical case series design was employed. The timeframe for data collection was extended from October 2019 through April 2022. This study used data from the Child Health and Mortality Prevention Surveillance (CHAMPS) study. CHAMPS utilizes high-quality data collection methodologies, including MITS, extensive pathogen testing, histopathology evaluation, clinical data extraction, and verbal autopsies [17, 18].

### Population and selection criteria

This study focused on the examination of deaths among neonates, infants, and children under the age of five that occurred within the health facilities situated in the HDSS area [16]. Stillbirths were excluded because antemortem diagnoses are not made in this population. There were three inclusion criteria for this study: Enrollment in CHAMPS, a MITS autopsy completed, and availability of dedicated clinical data for the deceased neonate, infant, or child within the healthcare facility where the death occurred. To maintain the integrity of the study and ensure the completeness and reliability of the data, cases, where antemortem clinical data was absent from the corresponding health facilities where the death occurred, were omitted from the study.

### Data collection procedures

A comprehensive and systematic approach was adopted for data collection, which included several sources of clinical data including documented history and physical examination, progress notes, nursing notes, order sheets, laboratory tests, radiological examinations, pathological examinations, and operation notes. The extraction of data was systematically carried out by qualified specialist physicians using a structured Excel sheet. Physicians who collected data were not involved in the clinical care of enrolled cases. To ensure the integrity of the data collected, all clinical data, including antemortem diagnoses, was verified independently by two additional physicians.

The antemortem clinical diagnoses were systematically extracted from all available clinical data sources, including documented history, physical examination findings, progress notes, nursing notes, order sheets, laboratory tests, radiological examinations, and, where appropriate, operative reports. The data extraction was conducted by qualified specialist physicians using a structured data collection tool. To ensure accuracy and completeness, the extracted antemortem diagnoses were independently verified by two additional physicians who were not involved in the clinical care of the cases.

While conducting MITS, postmortem biopsy needles were used to gather tissue samples, which underwent analysis using multiple testing methodologies. Specimens from MITS were utilized for histopathologic assessment, molecular analysis, and different microbial testing methodologies [19]. Samples underwent evaluation where multiplex molecular testing was conducted using TaqMan® Array Card (TAC) on fresh tissues to test for 126 pathogens. Furthermore, histopathology, special stains, immunohistochemistry (IHC), and molecular testing (PCR) were carried out on formalin-fixed paraffin-embedded (FFPE) tissues [19].

The highly sensitive detection of pathogens via TAC is possible on refrigerated MITS samples within a time window of three days following the occurrence of death. Moreover, gene Xpert testing, a fast molecular assay capable of detecting Mycobacterium tuberculosis and its rifampicin resistance was also used. Result agreement between TAC, IHC, and PCR methods varies, making it important to interpret all diagnostic tests in aggregate to establish overall case diagnoses and maximize the utility of these testing methods in MITS [19]. Additionally, clinical and epidemiologic data were considered, which has demonstrated enhanced interpretation of MITS findings [19].

The MITS process involved both local and international pathologists who reviewed histopathology results. A thorough assessment was conducted on both the obtained samples and locally produced slides within the CHAMPS network to ensure that they met all requirements [20]. The final cause of death was determined by a panel of local experts (the Determination of Cause of Death [DeCoDe] panel) that included obstetricians, pediatricians, pathologists, microbiologists, and epidemiologists [21] who reviewed all clinical, verbal autopsy, and histopathological data. The DeCoDe determined cause of death served as the reference standard for this study.

### Determination of diagnostic discrepancies

The antemortem clinical diagnosis and postmortem pathological diagnosis of the lung were compared for each case, and two physicians evaluated the result separately for concordance of diagnoses. These physicians were trained on the World Health Organization (WHO) classification approach of cases as concordant and discordant and assigning of reason for diagnostic discrepancy prior to commencement of the research. They used a structured Excel sheet to assign concordance and discordance and reason for all discrepancies. If there was a discrepancy between the physicians, a third physician was invited as an arbiter. The physicians classified the comparison of antemortem clinical diagnosis and postmortem causes of death into one of four categories according to predetermined categories: (1) diagnosed by both clinical and autopsy, (2) diagnosed by clinical and not by autopsy, (3) diagnosed by autopsy, not by clinical, and (4) not diagnosed by either. The reason for the diagnostic discrepancy was ascertained for each discrepant case by conducting an in-depth review of all available clinical data for each case in line with WHO guidelines on diagnostic discrepancy [22, 23].

The concordance analysis focused specifically on comparing antemortem clinical diagnoses with postmortem pathological diagnoses related to lung pathologies. Extrapulmonary findings identified during the postmortem evaluations were not considered in the concordance analysis, as they were not directly relevant to the primary objective of assessing diagnostic discrepancies in lung pathologies.

### Statistical analyses

Descriptive statistics were calculated and the McNemar’s test was utilized to identify any statistically significant discrepancies between antemortem and postmortem diagnoses. A p-value threshold of < 0.05 was set to determine statistical significance. In cases where discrepancies were identified, these were thoroughly discussed and pertinent recommendations for each discordant case were subsequently made. All analyses were conducted using Stata version 17.0 (StataCorp. 2021. Stata Statistical Software: Release 17. College Station, TX: StataCorp LLC).

## Results

### Sociodemographic characteristics of the participants

There was a total of 5,485 death notifications received by CHAMPS Ethiopia during the study period. Among those, 687 had MITS completed.

Among the cases enrolled for MITS, 612 (89.1%) lacked antemortem clinical data. This was primarily due to the majority being stillbirths (46%), with the remaining cases comprising deaths that occurred at home, on the way to a healthcare facility, or at the facility where medical records were lost or not documented. Seventy-five (10.9%) cases met inclusion criteria. 73% (*n* = 55) of participants were males, and 26.7% (*n* = 20) were females. Neonates aged 0–28 days at the time of death accounted for 86.7% (*n* = 65) of the study participants, with the majority of neonatal deaths occurring within the first week of delivery (70.7%, *n* = 53) (Table 1).

**Table 1:** Demographics of included cases from CHAMPS Ethiopia (2019 to 2022) (*N* = 75)

Characteristics	*N*	%
Gender		
Male	55	73.3
Female	20	26.7
**Age group at the time of death**		
Early neonates (≤ 24 h)	12	16.0
1–7 days	41	54.7
8 days-1 month	12	16.0
> 1 month-12 months	3	4.0
> 12 months to 59 months	7	9.3

A total of 206 different antemortem clinical diagnoses were recorded. The most common clinical diagnosis among the enrolled cases was early onset neonatal sepsis (30/206, 14.6%), followed by perinatal asphyxia (24/206, 11.7%) and preterm baby (23/206, 11.1%) (Table 2). Other common diagnoses included low birth weight (21/206,10.2%) and hypothermia (18/206, 8.7%).

**Table 2:** Percentages and frequencies of antemortem clinical diagnosis of cases included

Antemortem clinical diagnosis	*n*	Percent
Early onset neonatal sepsis	30	14.6%
Perinatal asphyxia	24	11.6%
Preterm baby	23	11.1%
Low birth weight	21	10.2%
Hypothermia	18	8.7%
Respiratory distress syndrome	11	5.3%
Pneumonia	6	3.0%
Meconium aspiration syndrome	6	3.0%
Apnea of the newborn	6	3.0%
Hyaline membrane disease	6	3.0%
Meningitis	6	3.0%
Severe acute malnutrition	5	2.4%
Hypoglycemia	4	2.0%
Late onset neonatal sepsis	3	1.4%
Hydrocephalus	3	1.4%
Hypovolemic shock	3	1.4%
Severe anemia	2	1.0%
Infantile hypertrophic pyloric stenosis	2	1.0%
Spina bifida	2	1.0%
Measles	1	0.5%
Autosomal recessive polycystic kidney disease	1	0.5%
Acute kidney injury	1	0.5%
Scabies	1	0.5%
Caput Succedaneum	1	0.5%
Ludwig angina	1	0.5%
Phocomelia	1	0.5%
Nephrotic syndrome	1	0.5%
Neonatal hyperbilirubinemia	1	0.5%
Opitz Trigonocephaly Syndrome	1	0.5%
Right inguinal hernia	1	0.5%
portal hypertension	1	0.5%
Chronic liver disease	1	0.5%
Spontaneous bacterial peritonitis	1	0.5%
Acute gastro enteritis	1	0.5%
Cephalic hematoma	1	0.5%
Febrile seizure	1	0.5%
COVID-19	1	0.5%
Hemorrhagic disease of the newborn	1	0.5%
Deep intravascular coagulation	1	0.5%
Subgaleal hemorrhage	1	0.5%
Paraplegia	1	0.5%
Intra cranial hemorrhage	1	0.5%
Chiari malformation	1	0.5%
Oral thrush	1	0.5%
Total	206	100%

There was a total of 147 postmortem pathologic diagnoses for the immediate, underlying, and comorbid conditions of the 75 cases (Table 3). The most common postmortem pathologic diagnosis observed was extramedullary hematopoiesis, (32/147, 21.7%), followed by hyaline membrane disease (17/147, 11.6%) and sepsis (13/147, 8.8%). Pneumonia and meconium aspiration syndrome also each contributed to 8.8% of the total cases, with 13 instances each.

**Table 3:** Percentages and frequencies of postmortem diagnoses of included cases

Postmortem Pathologic Diagnoses	*n*	Percent
Extramedullary hematopoiesis	32	21.7%
Hyaline membrane disease	17	11.6%
Sepsis	13	8.8%
Pneumonia	13	8.8%
Meconium aspiration syndrome	12	8.2%
Intrauterine fetal distress	12	8.2%
Interstitial pneumonitis	11	7.5%
Steatosis	8	5.4%
Meningitis	5	3.4%
Bronchopneumonia	4	2.7%
Preterm lung features	4	2.7%
Sinusoidal leukocytosis	2	1.4%
Pulmonary Hemorrhage	2	1.4%
Centrilobular necrosis	2	1.4%
Granulomatous inflammation	1	0.7%
Hypoxic ischemic encephalopathy	1	0.7%
Intravascular leukocytosis in liver	1	0.7%
Spina bifida	1	0.7%
Congenital limb defect	1	0.7%
Hepatic necrosis	1	0.7%
Fibro purulent pneumonitis	1	0.7%
Hemorrhagic pulmonary edema	1	0.7%
Respiratory distress syndrome	1	0.7%
Interalveolar hemorrhage	1	0.7%
Total	147	100%

### Antemortem clinical and postmortem autopsy diagnosis concordance

Of the 75 included cases included, 52.0% (*n* = 39) were accurately diagnosed by antemortem clinical diagnosis compared to postmortem diagnoses (Table 4). There was a diagnostic discordance in 37.3% (*n* = 28) cases. Overall, there was a 35% (95% CI: 20%, 47%) diagnostic discrepancy between antemortem and postmortem diagnoses (*p* < 0.0001) (Table 5).

**Table 4:** Concordance and discordance of antemortem clinical and postmortem pathologic diagnosis among cases included in CHAMPS study from 2019–2022

	*n*	%
Concordant antemortem and postmortem diagnoses	39	52.0
Antemortem diagnosis discordant with postmortem	28	37.0
Antemortem diagnosis with no postmortem diagnosis	3	4.0
Missing antemortem and postmortem diagnoses	5	7.0

**Table 5:** The proportion of discrepancy between antemortem clinical and postmortem autopsy diagnosis of lung and McNemar’s test result among cases included in CHAMPS research from 2019–2022

Antemortem clinical diagnosis	Postmortem autopsy diagnosis			
	Yes	No	Total	
Yes	39	3	42	
No	28	5	33	
Total	67	8	75	
The proportion of postmortem autopsy diagnoses with certain diagnosis in comparison with the final DeCoDe diagnosis			89%	
The proportion of antemortem clinical diagnosis with certain diagnosis in comparison with the final DeCoDe diagnosis			54%	
Discrepancy			35% (95% CI:20-47%)	
P-value			0.0001	

There were 31 (41.3%) cases in which the clinical antemortem and postmortem pathology causes of death were discordant. Three (4.0%) were detected by clinical diagnosis but not by pathologic diagnosis, and 28 (37.3%) were detected by pathologic diagnosis but not by clinical diagnosis.

### Reasons for diagnostic discrepancies

The majority of diagnostic discrepancies (71%, *n* = 44/62) were attributed to case analysis problems and deficiencies in consultation and teamwork. Other issues including documentation errors, unavailability of diagnostic investigations, communication gaps, limited access to health facilities, and existing work culture were also identified (Table 5). The analysis of reasons for the diagnostic gap in cases undiagnosed by antemortem clinical diagnosis and diagnosed by postmortem autopsy are elaborated in Table 6 and each reason for the diagnostic discrepancy is described with illustrative case as an example.

**Table 6:** Reasons for the diagnostic discrepancy among included cases

Reason for Diagnostic Discrepancy	*n*	Description	Illustrative Case Example	Result
Case Analysis Problem (Knowledge gap or comprehension problem)	22 (35.5%)	This category refers to discrepancies due to a lack of knowledge or understanding in disease recognition or clinical presentation.	Study ID: CPCS009 is a female neonate with severe respiratory issues and an open neural tube defect. Despite clear signs of a potential pathologic lung problem and an established risk of sepsis with open neural tube defect, sepsis and meningitis were not considered in the diagnosis.	Missed diagnosis
Problem in Consultation and Teamwork	22 (35.5%)	These discrepancies occur due to issues in case analysis, possibly preventable with improved consultation and teamwork, particularly with experienced staff or pediatricians.	Study ID: CPCS025 is a two-year-old male child who initially presented with abdominal swelling and later developed respiratory distress. Despite obvious respiratory symptoms and clinical conditions indicative of potential pneumonia, an antemortem diagnosis of lung pathologies was not considered.	Missed diagnosis
Documentation Problem	6 (10.0%)	This category refers to discrepancies due to gaps in documentation of patient’s condition, delivery details, referral, and progress.	Study ID: CPCS064 is a neonate with unknown weight and Apgar score who developed difficulty breathing immediately after birth. The absence of documented labor duration, Apgar score, meconium staining, resuscitation attempts, and suspected diagnosis at delivery, contributed to the missed diagnosis by clinicians at Haramaya Hospital.	Missed diagnosis
Unavailability of Diagnostic Investigations	5 (8.0%)	This category refers to discrepancies due to a lack of sufficient diagnostic investigation setups in health facilities.	Study ID: CPCS018 is a term neonate presenting with respiratory distress and unusual facial features. Despite atypical presentation for her gestational age, a diagnosis of hyaline membrane disease (HMD) was not considered due to a lack of available diagnostic investigations such as portable chest x-ray or arterial blood gas analysis.	Missed diagnosis
Communication Gap Between Health Facilities	4 (6.0%)	This category refers to discrepancies due to gaps in communication between health facilities during patient referral. Lack of information about the history, physical examination, investigation, diagnosis, and treatment of patients can lead to diagnostic discrepancy.	Study ID: CPCS035 is a newborn twin (Twin B) who weighed 1000 g and presented with signs of distress. Despite the twin pregnancy, labor complications, and risk of twin-twin transfusion syndrome, there was no partograph or documentation on the progress of labor on the referral sheet. The condition of meconium aspiration syndrome, a potential risk in such cases, was also not included in the referral diagnosis, leading to a missed diagnosis. This case indicates a communication gap between the private clinic and Hiwot Fana Hospital.	Missed diagnosis
Communication Gap Between Health Facilities	4 (6.0%)	This category refers to discrepancies due to gaps in communication between health facilities during patient referral. Lack of information about the history, physical examination, investigation, diagnosis, and treatment of patients can lead to diagnostic discrepancy.	Study ID: CPCS035 is a newborn twin (Twin B) who weighed 1000 g and presented with signs of distress. Despite the twin pregnancy, labor complications, and risk of twin-twin transfusion syndrome, there was no partograph or documentation on the progress of labor on the referral sheet. The condition of meconium aspiration syndrome, a potential risk in such cases, was also not included in the referral diagnosis, leading to a missed diagnosis.	Missed diagnosis
Access to Health Facility	2 (3.0%)	This category refers to discrepancies due to the lack of access to advanced healthcare facilities, often resulting from geographical distance and referral procedures.	Study ID: CPCS029 is a 3-week-old male neonate who died at home without receiving healthcare services. The child had signs of measles but was unable to access medical help. Postmortem autopsy revealed pneumonia, which is a common cause of death in children with measles.	Missed diagnosis
Work Culture	1 (2.0%)	Diagnostic discrepancies resulting from work culture are tied to concerns about accountability, which may lead to underreporting or misreporting of medical conditions. A supportive work environment that encourages learning from past mistakes is vital to address this issue.	Study ID: CPCS005 involves a child who was brought from a private orphanage to Hiwot Fana hospital, severely ill and with a three-day history of diarrhea. The child was documented as ‘death on arrival’, with no other documentation about the condition. Postmortem autopsy indicated sepsis.	Missed diagnosis

## Discussion

Our investigation elucidated the accuracy of antemortem clinical diagnoses and their alignment with postmortem pathologic diagnoses in childhood mortality cases in regions with high childhood mortality in Ethiopia. This investigation revealed that 37.3% of cases had discrepant antemortem clinical diagnoses and postmortem pathologic diagnoses among children with positive lung pathology findings. Most diagnostic errors were attributed to gaps in clinical knowledge and a lack of collaboration and specialist consultation.

The degree of diagnostic discrepancy observed in this investigation aligns with those reported in prior studies. The rate of diagnostic discrepancy manifested in the current study also corresponds with global rates. However, there appears to be a pronounced pattern of higher diagnostic discrepancies in resource-constrained nations relative to those in high-income settings [21, 24]. This highlights an important systemic issue in low- and middle-income countries, accentuating the diagnostic challenges posed by resource limitations. For instance, research conducted in Mozambique revealed a diagnostic discrepancy rate of 63% [25], one of the highest reported in the sub-Saharan African region. Contrasting this, a study in Germany demonstrated a progressive decline in diagnostic discrepancy rates over a ten-year period, from 43% in 1998 to 27% in 2008 [26]. This downward trend suggests a steady advancement in diagnostic capabilities over time and underscores the disparities in diagnostic certainty across varied socioeconomic contexts given higher rates of diagnostic errors in sub-Saharan Africa.

Furthermore, in the Netherlands, a mere 35% of cases demonstrated complete accuracy in the clinical determination of the cause of death and a Brazilian investigation indicated an agreement between clinical and pathological diagnoses in 58% of cases [25, 27]. The patterns unearthed in these studies imply that, even within the confines of high-income nations, significant diagnostic discrepancies endure. These observations suggest an urgent need to address prevalent diagnostic errors, regardless of the country’s economic status. This need is particularly pronounced in countries characterized by limited resources [27–29]. Concerted efforts towards the enhancement of clinical diagnostic procedures are needed to reduce antemortem diagnostic errors, ensuring the provision of more accurate diagnoses and corresponding treatments to reduce mortality [27, 29, 30].

The principal contributor to diagnostic discrepancies in our study was issues pertaining to case analysis, accounting for more than one in three of all instances. These problems often originate from a deficiency in knowledge concerning disease pathology, clinical presentation, and interpretation of commonplace scenarios in routine practice [31]. This highlights the imperative for continuous professional education and dissemination of the latest clinical knowledge, enabling healthcare professionals to stay abreast of advancements in their fields. Similarly, issues related to consultation and teamwork constituted another 35.5% of the discrepancies. Deficient communication amongst healthcare professionals, limited involvement of senior clinicians, and ineffective teamwork were identified as key factors precipitating these challenges, underscoring the crucial role of proficient communication and collaboration within healthcare settings in Ethiopia [32].

Furthermore, gaps in documentation contributed to 10% of diagnostic discrepancies. Inaccurate or incomplete patient records not only disrupt the continuity of patient care but also impede inter-professional communication, thereby leading to avoidable medical errors. This indicates an urgent need for health systems to refine their documentation practices, ensure meticulous maintenance of patient records [33]. The unavailability of diagnostic investigations factored into 8% of the discrepancies. In low- and middle-income countries, a lack of access to diagnostic tools poses a significant problem [34, 35], emphasizing the need to bolster healthcare infrastructure, particularly regarding diagnostic modalities. Previous studies have described numerous contributing factors to diagnostic discrepancies, with a significant emphasis on the influence of systemic, cognitive, and logistical components [36]. Lapses in clinical diagnoses may potentially precipitate adverse patient outcomes. Prior studies suggest the causes of diagnostic discrepancies are often multifaceted [36]. Hence, a comprehensive and multidimensional approach is imperative to mitigate such diagnostic discrepancies.

This investigation emphasizes the importance of sustained monitoring of clinical diagnostic accuracy and calls for systemic enhancements in various facets of healthcare practice. The highlighted areas of concern necessitate targeted interventions and further research to mitigate diagnostic discrepancies and improve patient outcomes. Strengths of the study include the use of postmortem autopsies as the benchmark for cause-of-death determination, facilitating precise comparisons between antemortem clinical and postmortem pathological diagnoses of lung pathologies. The first of its kind, this study provides an initial investigation of clinicopathologic diagnosis discrepancies in Ethiopia among young children, laying the groundwork for further research to elucidate reasons for diagnostic errors and efforts to mediate these errors. The comprehensive dataset amplifies the study’s validity. The meticulous evaluation performed by two senior physicians, with a third involved in the event of disagreement, ensures rigor in the study.

However, some limitations merit consideration. The sole focus on lung pathologies potentially restricts the study’s insights into other body systems. The modest sample size reduces statistical power and restricts the application of advanced statistical methods. Being a case series study, the lack of a control group inhibits direct comparisons and controls for confounding variables. However, conducting lung biopsies on living children in which such a procedure is not indicated would be unethical.

There was a high proportion of cases that did not have antemortem clinical diagnoses available so were excluded from our analyses, which may reflect inadequate record keeping in clinical care. This could potentially lead to an underestimation of the prevalence of diagnostic errors.

It is important to note that our study did not analyze the potential relationship between the level of healthcare facilities (e.g., primary care centers, district hospitals, referral hospitals) and the occurrence of diagnostic discrepancies. Exploring this aspect could provide valuable insights into the specific challenges faced by healthcare providers at different levels of the healthcare system. Future research should consider examining the distribution of diagnostic discrepancies across various levels of healthcare facilities to inform targeted interventions and resource allocation strategies aimed at reducing diagnostic errors.

## Conclusions

There was substantial discordance between antemortem clinical diagnoses and postmortem autopsy results in this study, which points to a critical need for comprehensive reform in several facets of clinical practice in Ethiopia. The identified contributing factors – including gaps in clinical knowledge and understanding, limitations in teamwork and consultation processes, problems related to patient documentation, and scarcity of diagnostic investigations– illustrate complex, multifaceted issues that contribute to diagnostic errors. The direct implication of these findings is an urgent call for robust, systemic interventions to enhance diagnostic accuracy and patient care outcomes. It is paramount that future efforts focus on reducing these discrepancies through improved provider pre-service and in-service education, communication, and resource allocation within the healthcare sector.

## Data Availability

The datasets generated and analyzed during the current study are not publicly available due to the sensitive nature of the information. However, they are available from the principal investigators upon reasonable request and with appropriate ethical approval.

## Abbreviations

APGAR: Appearance, Pulse, Grimace, Activity, and Respiration
CDA: Complete diagnostic autopsy
CHAMPS: Child health and mortality prevention surveillance
DeCoDe: Deciding cause of death
WHO: World Health Organization
HDSS: Health and demographic surveillance site
HMD: Hyaline membrane disease
